# Respiratory Syncytial Virus Nonstructural Protein 1 Promotes 5-Lipoxygenase via miR-19a-3p

**DOI:** 10.1155/2022/4086710

**Published:** 2022-05-20

**Authors:** Mei Zheng, Panpan Fan, Pu Yang, Junwen Zheng, Dongchi Zhao

**Affiliations:** Department of Pediatrics, Children's Digital Health and Data Center, Zhongnan Hospital of Wuhan University, Wuhan, China

## Abstract

**Background:**

Respiratory syncytial virus (RSV) infection can regulate the expression of a wide range of noncoding microRNAs (miRNAs), in which mir-19a-3p can participate in airway inflammatory response by regulating 5-lipoxygenase (5-LO) pathway. RSV nonstructural protein (NS) 1 is involved in the airway hyperresponsiveness during RSV infection.

**Methods:**

The expression levels of miR-19a-3p and inflammatory signaling-related indicators were detected using quantitative real-time PCR and western blot analyses on the A549 cells transfected with NS1 expression plasmids (pNS1). The 5-LO-mediated inflammatory signaling pathway was assessed when the miR-19a-3p or 5-LO was inhibited.

**Results:**

The immunofluorescence analysis showed that the plasmid-mediated NS1 protein was observed in both the cytoplasm and nucleus. The expression level of miR-19a-3p was significantly upregulated in the pNS1 or RSV-treated cells, which was reversed by the NS1 small interfering RNA. In addition, pNS1 also upregulated the expression of 5-LO, interleukin-5 (IL-5), and leukotriene B_4_ (LTB_4_), which was also significantly inhibited by the miR-19a-3p antagonists. The 5-LO inhibitor MK886 prevented the increase in the expression level of IL-5 induced by pNS1.

**Conclusions:**

These results suggested that the RSV NS1 might play an important role in the pathogenesis of RSV by activating the 5-LO and subsequent inflammatory cytokines through miR-19a-3p.

## 1. Introduction

Respiratory syncytial virus (RSV) is the most common pathogen of the respiratory tract in infants and children under 2 years of age and is also closely related to the occurrence of asthma and wheezing in children [1]. It is a single-strand negative RNA virus, which belongs to the genus *Pneumovirus* in the family *Paramyxoviridae*. RSV encodes 11 proteins, including the two nonstructural (NS) proteins (NS1 and NS2). Despite their absence in mature virions, the NS proteins are the most abundantly transcribed viral genes. Functionally, the RSV NS1 is involved in viral replication, transcription, and metabolic functions [2, 3]. The viral load and airway inflammation in the RSV-infected mice decreased significantly after the NS1 inhibition [4]. Similarly, the RSV mutants, lacking NS1, also showed significant attenuation and immunogenicity in chimpanzees [5]. NS1 protein could act as an independent pathogenic factor in mice and cause pathological changes in their lungs [6]. Additionally, the NS1 proteins are also involved in inflammation and airway hyperresponsiveness (AHR) during RSV infection [7].

Leukotrienes (LTs) are produced by leukocytes, bronchial epithelial cells, and fibroblasts at the initial stages of an acute inflammatory response and act predominantly as proinflammatory lipid mediators. 5-Lipoxygenase (5-LO) is a key enzyme, which catalyzes the transformation of arachidonic acid into inflammatory LTs [8]. 5-LO is produced by various cells, including neutrophils, eosinophils, monocytes/macrophages, dendritic cells, mast cells, and lymphocytes [9], and converts arachidonic acid into leukotriene A_4_ (LTA_4_). LTA_4_ is then transformed into leukotriene B_4_ (LTB_4_) or leukotriene C_4_ (LTC_4_), which are exported from the cell [10]. LTs, such as LTB_4_, are the pivotal inflammatory mediators during an asthma attack or wheezing. LTB_4_ serves as a potent inflammatory factor and is mediated through a high-affinity LTB_4_ receptor-1 (BLT1) present on the target cells. For instance, the LTB_4_ attracts neutrophils, monocytes, and lymphocytes to the site of inflammation in the airways and stimulates the secretion of mucus, elastase, and superoxide radicals, as well as that of inflammatory cytokines, which lead to the formation of edema by increasing vascular permeability and plasma leakage at the site of inflammation. In clinical practices, the LT receptor blockers are widely used for the treatment of acute wheezing caused by RSV infection. These studies indicated that the 5-LO-LT pathway might be involved in the pathogenesis of RSV infection. However, the mechanisms of how RSV regulates the expression of 5-LO and LTs and whether the RSV infection is mediated by NS1 or not are still unclear.

MicroRNAs (miRNAs) are small noncoding RNAs, which are composed of 20-24 nucleotides. miRNAs can induce the degradation of target mRNAs or inhibit their translation and are widely involved in the regulation of various physiological and pathological processes in cells [11, 12]. They also regulate viral infection by altering the host's response to inflammatory cells, immune cells, and airway epithelial cells [13, 14]. After RSV infection, multiple miRNAs have been confirmed to be abnormally expressed *in vivo* or *in vitro*. The RSV infection of A549 cells affected a set of miRNAs, particularly the *let-7f* expression [15]. Recombinant RSV, lacking the *NS1* gene, could induce the miR-24 expression [16], while the inhibition of *NS1* and *NS2* genes resulted in elevated *let-7i* and miR-30b expression [17]. In our previous study, it was demonstrated that the RSV infection could induce a variety of differentially expressed miRNAs in infants [18]. Among them, miR-19a-3p has been shown to regulate the activity of 5-LO [19]. Considering the important role of 5-LO in LT production and that of LTs in RSV-induced wheezing, it could be hypothesized that the activation of miR-19a-3p and 5-LO by RSV NS1 might be a virulence mechanism employed by RSV.

In order to test the hypothesis, an RSV NS1-expressing plasmid (pNS1) was transfected into the A549 cell to study the effects of RSV NS1 on the expression of inflammatory cytokines mediated by the miR-19a-3p and 5-LO pathway as well as to explore the miRNA-related mechanism of cellular inflammation caused by NS1.

## 2. Materials and Methods

### 2.1. Cells, Plasmids, and Virus

Type II human lung epithelial cell line A549 was obtained from the American Type Culture Collection (ATCC, Manassas, VA, USA) and cultured in a humidified incubator at 37°C with 5% CO_2_ concentration. A high-glucose Dulbecco's modified Eagle medium (DMEM) (Gibco, Grand Island, NY, USA) supplemented with 10% fetal bovine serum (FBS) (Gibco) was used for the culturing of A549 cells. The cell culture was passaged when it grew to a dense monolayer. pNS1 was constructed as previously described [20] and stored as frozen glycerol stocks. The RSV A2 strain was originally provided by Professor Pan Zishu from the Academy of Life Science, Wuhan University, China, and was stored at -80°C.

### 2.2. Plasmid Transfection and miRNA Inhibition

Plasmid transfection and miRNA inhibition were performed using Lipofectamine 3000 following the manufacturer's instructions (Invitrogen, Carlsbad, CA, USA). Briefly, the cells were grown to 60~70% confluence in a six-well plate before transfection. For the plasmid transfection, a mixture of plasmid DNA (2.5 *μ*g), Lipofectamine 3000 (7.5 *μ*L), and P3000 (5 *μ*L) in 250 *μ*L reduced serum medium (Opti-Minimal Essential Medium, Opti-MEM, Gibco) was added to each well. Small interfering RNAs (siRNAs), targeting the RSV NS1 (siNS1), or negative control siRNAs (siNC) were used for the siRNA transfection. For this purpose, a mixture of siRNA (75 pmol) and Lipofectamine 3000 (7.5 *μ*L) in an Opti-MEM medium (250 *μ*L) was added to each well. For the inhibition of mRNA, the cells were treated with a mixture of miR-19a-3p antagomir (100 nM) and Lipofectamine 3000 (125 *μ*L) for 12 h before pNS1 transfection. The transfected cells were maintained at 37°C and 5% CO_2_ concentration in a humidified incubator and were harvested at specific time points.

### 2.3. Virus Inoculation

The A549 cells were grown to 60~70% confluence and were pretreated with siNC or siNS1 for 6 h before RSV inoculation, as described above. For a six-well plate, 500 *μ*L RSV (10^5^ PFU/mL) was added to each well and incubated for 2 h at 37°C. Then, the viral solution was replaced with 2% FBS-containing DMEM. The plates were then incubated at 37°C, and the cells were harvested for further experiments at the indicated time points.

### 2.4. Inhibition of 5-LO

At the indicated time points before plasmid injection, the cells were pretreated with 20 *μ*M of the 5-LO inhibitor MK886 (MCE, New Jersey, USA) [21] to inhibit the activation of 5-LO. The cell viability assay was performed using a Cell Counting Kit-8 (CCK-8, Meilunbio, Dalian, China) following the manufacturer's protocol in order to detect the cytotoxic effects of MK886 on the cells.

### 2.5. Total RNA Extraction and Reverse Transcription-Real-Time Quantitative PCR (RT-qPCR)

The total RNA was extracted from the A549 cells using TRIzol reagent (Invitrogen, CA, USA) following the manufacturer's protocol. A total of 1 *μ*g of the extracted RNA was reverse-transcribed into a 20 *μ*L reaction mixture using a miDETECT A Track miRNA RT-qPCR Kit (RiboBio, Guangzhou, China). RT-qPCR was conducted using 2 × SYBR Green Mix (RiboBio, Guangzhou, China) according to the manufacturer's instructions. The 20 *μ*L PCR reaction mixture contained 2 *μ*L of cDNA product, 10 *μ*L of 2 × SYBR Green Mix, and 10 *μ*M of each forward and reverse primers. U6 small nuclear RNA was used as a reference control for the miRNA expression. Primers for the human miR-19a-3p and *U6* genes were purchased from RiboBio (Guangzhou, China). The primer sequences were as follows: human *5-LO* gene (forward 5′-GGTGGATTCATACGACGTGACT-3′, reverse 5′-GGTAAATCCTTGTGGCATTTGG-3′), human *IL-5* gene (forward 5′-CTTTCAGGGAATAGGCACAC-3′, reverse 5′-GTTTACTCTCCGTCTTTCTTC-3′), and human *GAPDH* gene (forward 5′-TGATGACATCAAGAAGGTGG-3′, reverse 5′-TTACTCCTTGGAGGCCTAGT-3′). The relative gene expression was determined using the 2^-*ΔΔ*Ct^ method, where the Ct values of *U6* and *GAPDH* genes were used for normalization purposes.

### 2.6. Western Blot Analyses

Total protein contents were extracted from the A549 cells using RIPA lysis buffer, and their concentrations were determined using a bicinchoninic acid assay (Beyotime, Shanghai, China). Western blot was performed as previously described [22]. Since the FLAG tag was cloned into the pNS1, an antibody against the FLAG tag was used to detect NS1-FLAG. *β*-Actin protein was used as an internal reference. The primary antibodies against the FLAG tag and *β*-actin proteins were purchased from Cell Signaling Technology (CST, Boston, USA), and those of 5-LO and IL-5 were purchased from Proteintech (Wuhan, China). All the primary antibodies were used at a dilution of 1 : 1000 and incubated overnight at 4°C. The horseradish peroxidase- (HRP-) conjugated secondary antibody (1 : 5000 diluted, Servicebio, Wuhan, China) was incubated for 1 h at room temperature. Then, the blots were visualized using an enhanced chemiluminescence (ECL) detection kit (Servicebio, China) and detected using a chemiluminescence imaging system (Tanon, Shanghai, China). The results were quantified using the ImageJ Launcher (version 1.8.0).

### 2.7. Immunofluorescence

The A549 cells were grown on Nunc™ Petri dish (Thermo Fisher, Waltham, MA, USA) and then fixed with 4% paraformaldehyde in phosphate-buffered saline (PBS) buffer for 30 min. After washing with PBS, the cells were permeabilized with 0.1% Triton X-100 in PBS buffer for 15 min and then blocked with 1% bovine serum albumin (BSA) (Thermo Fisher, Waltham, MA, USA) for 30 min. After blocking, the cells were incubated (4°C overnight) with rabbit anti-FLAG antibody (Cell Signaling Technology, Boston, USA), which was used as a primary antibody. The cells were then washed with PBS and incubated with fluorescein isothiocyanate- (FITC-) conjugated fluorescent antibody (Bioss, Beijing, China) for 1 h at room temperature. The nuclei of the cells were counterstained with 4,6-diamidino-2-phenylindole (DAPI) (Bioss, Beijing, China) for 5 min and visualized using a confocal laser scanning microscope (Leica, Germany).

### 2.8. LTB_4_ Measurement

The cell supernatants were collected as previously described [23]. Briefly, approximately 10^6^ cells were suspended in 1 mL PBS and incubated on ice for 30 min. Then, the cells were centrifuged at 400 g for 5 min, resuspended in DMEM (serum-free), and incubated at 37°C for 30 min. The secretion of LTB_4_ was stopped by adding cold PBS before the centrifugation of cells and collection of supernatants. Then, the LTB_4_ concentrations were measured using an enzyme-linked immunosorbent assay kit (R&D Systems, Minneapolis, USA).

### 2.9. Statistics

All the experiments were carried out in triplicates. The results are expressed as the mean ± SD and analyzed using the GraphPad Prism v6.0 Software. Differences between the means were analyzed using a paired Student's *t*-test or one-way analysis of variance. The results were considered statistically significant at a *P* value of less than 0.05.

## 3. Results

### 3.1. RSV NS1 Upregulated the miR-19a-3p Expression in A549 Cells

**Figure 1:**
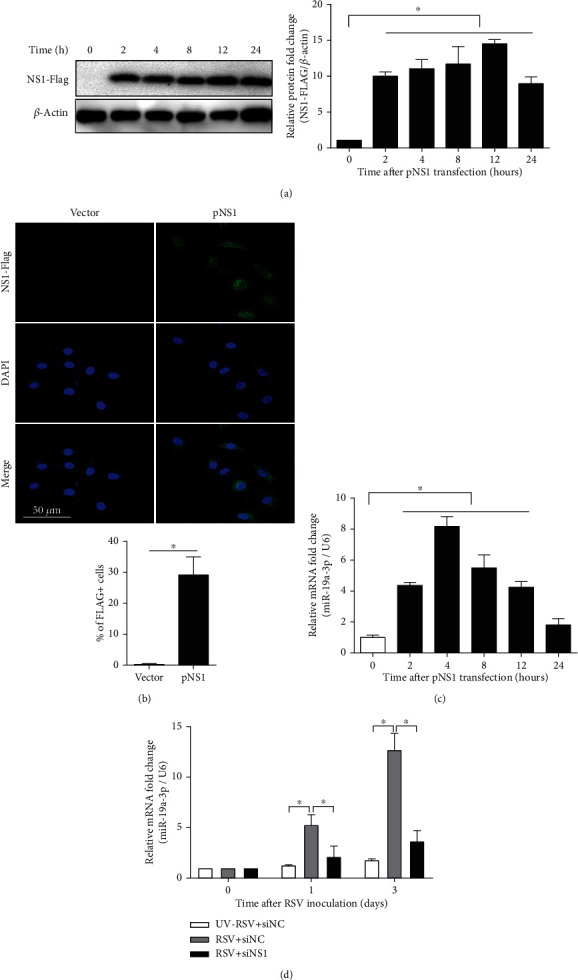
RSV NS1 protein upregulates the expression of miR-19a-3p in A549 cells. (a) Western blot analysis of NS1-FLAG protein in A549 cells transfected with pNS1. Anti-FLAG antibodies were used to assess for the presence of FLAG-tagged NS1. *β*-Actin was used as an internal control. (b) The expression levels of miR-19a-3p mRNA at different time points after pNS1 transfection. U6 RNA was used as an internal control. (c) Localization of NS1 (green) in A549 cells transfected with pNS1 was analyzed by immunofluorescence confocal microscopy at four hours after transfection. Anti-FLAG antibodies were used to assess for the presence of FLAG-tagged NS1. Nuclei were counterstained with DAPI (blue). Representative images were shown. (d) The expression levels of miR-19a-3p mRNA in A549 cells at different time points after RSV inoculation. Monolayers of A549 cells were infected for 72 hours with ultraviolet-inactivated RSV or live RSV. Cells were pretreated with siNC or siNS1 for 6 hours prior to RSV inoculation. U6 RNA was used as an internal control. Data are expressed as mean ± SD (*n* = 3 technical replicates). The experiment was performed three times with similar results, and one representative experiment is shown. ^∗^*P* < 0.05 vs. 0 h. NS1: nonstructural protein 1; pNS1: nonstructural protein 1 expressing plasmid; DAPI: 4′,6-diamidino-2-phenylindole; RSV: respiratory syncytial virus; UV-RSV: ultraviolet-inactivated RSV; siNC: negative control siRNA; siNS1: RSV NS1 siRNA.

In order to determine whether NS1 could regulate the expression of miR-19a-3p in epithelial cells, A549 cells were transfected with pNS1. The NS1 protein was significantly expressed 2 h after transfection and reached the peak at about 12 h (Figure 1(a)). The immunofluorescence detection at 4 h posttransfection showed that the NS1-FLAG was observed in both the cytoplasm and nucleus of the A549 cells (Figure 1(b)). The expression of miR-19a-3p mRNA was upregulated at 2 h, reached the maximum at 4 h, and lasted for 12 h after pNS1 transfection (Figure 1(c)). In order to verify the effects of RSV NS1 on miR-19a-3p expression, the A549 cells were pretreated with siNS1 for 6 h and then inoculated with RSV. As compared to ultraviolet inactivated RSV (UV-RSV), the RSV significantly increased the expression levels of miR-19a-3p, which was significantly inhibited by the application of siNS1 (Figure 1(d)). These results suggested that RSV NS1 protein could enter the nuclei of epithelial cells and enhance the expression of miR-19a-3p by influencing the nuclear transcription.

### 3.2. pNS1 Promoted the IL-5 and 5-LO Expression

**Figure 2:**
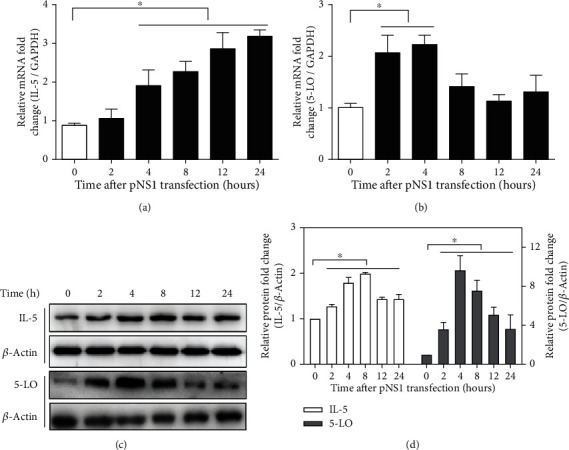
RSV NS1 protein promoted inflammatory factor production and increased 5-lipoxygenase (5-LO) expression. (a, b) The expression levels of IL-5 and 5-LO mRNA in A549 cells transfected with pNS1 were detected by RT-qPCR. GADPH was used as an internal control. (c) Western blot analysis of 5-LO protein and IL-5 protein in A549 cells transfected with pNS1. *β*-Actin served as an internal control. (d, e) Relative quantitative analysis of 5-LO protein and IL-5 protein, respectively. Data are expressed as mean ± SD (*n* = 3 technical replicates). The experiment was performed three times with similar results, and one representative experiment is shown. ^∗^*P* < 0.5 vs. 0 h. 5-LO: 5-lipoxygenase; IL-5: interleukin-5; pNS1: nonstructural protein 1 expressing plasmid.

In order to investigate whether pNS1 could affect the expression of IL-5 and 5-LO, the pNS1-transfected A549 cells were collected at different time points to harvest total protein and total RNA. As shown in Figure 2(a), the expression levels of IL-5 mRNA gradually increased and reached the maximum at 24 h of pNS1 transfection as compared to that in the vector control. The expression level of 5-LO mRNA sharply increased and reached the maximum at 2-4 h of pNS1 transfection (Figure 2(b)). Similarly, western blot results also showed that the protein level of IL-5 protein increased after pNS1 transfection and reached its maximum at about 8 h. The 5-LO protein level increased at 2 h (Figures 2(c) and 2(d)) and had a significant change at 4-12 h (*P* < 0.05).

### 3.3. Inhibition of miR-19a-3p Attenuated the 5-LO and IL-5 Expression Mediated by pNS1

**Figure 3:**
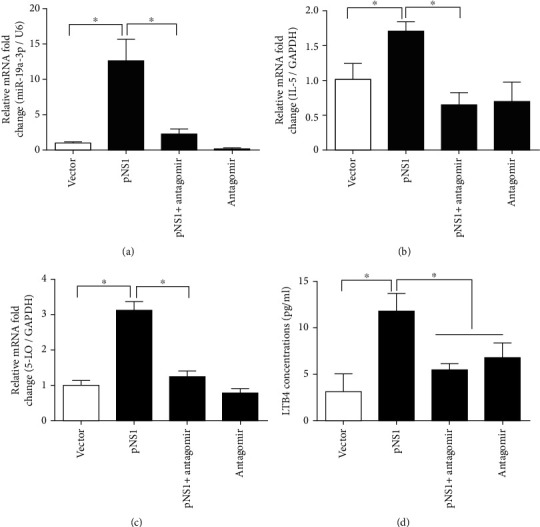
Inhibition of miR-19a-3p attenuated the promotive effects of NS1 on 5-lipoxygenase (5-LO) and interleukin-5 (IL-5) expression. (a–c) The mRNA expression levels of miR-19a-3p, IL-5, and 5-LO in A549 cells transfected with pNS1 were detected by RT-qPCR four hours after transfection. U6 RNA or GADPH was used as internal control, respectively. (d) Concentrations of leukotriene B4 in the supernatants of A549 cells were detected by an enzyme-linked immunosorbent assay. Data are expressed as mean ± SD (*n* = 3 technical replicates). The experiment was performed three times with similar results, and one representative experiment is shown. ^∗^*P* < 0.5 vs. control group. LTB_4_: leukotriene B4; 5-LO: 5-lipoxygenase; IL-5: interleukin-5; pNS1: nonstructural protein 1 expressing plasmid.

To understand the correlations between miR-19a-3p and 5-LO and IL-5 expression levels after pNS1 transfection, the A549 cells were pretreated with a miR-19a-3p antagomir for 12 h before transfection with pNS1. Then, the treated cells were harvested at 4 h after pNS1 transfection for the analysis of mRNA and protein expression. The results showed that, in the presence of miR-19a-3p antagomir, both the baseline expression and pNS1-mediated overexpression of miR-19a-3p were significantly inhibited with an inhibition efficiency of 81.7% and 83.2%, respectively (Figure 3(a)). As compared to that in the control group, the expression level of IL-5 (Figure 3(b)) was significantly inhibited by miR-19a-3p antagomir. The pNS1-mediated upregulation of 5-LO mRNA (Figure 3(c)) was reduced by the inhibitory effect of miR-19a-3p. In addition, the LTB_4_ concentrations were also significantly reduced by the miR-19a-3p antagomir in the pNS1-transfected cells (Figure 3(d)).

### 3.4. Inhibition of 5-LO Attenuated the Promotive Effects of pNS1 on IL-5 and LTB_4_

**Figure 4:**
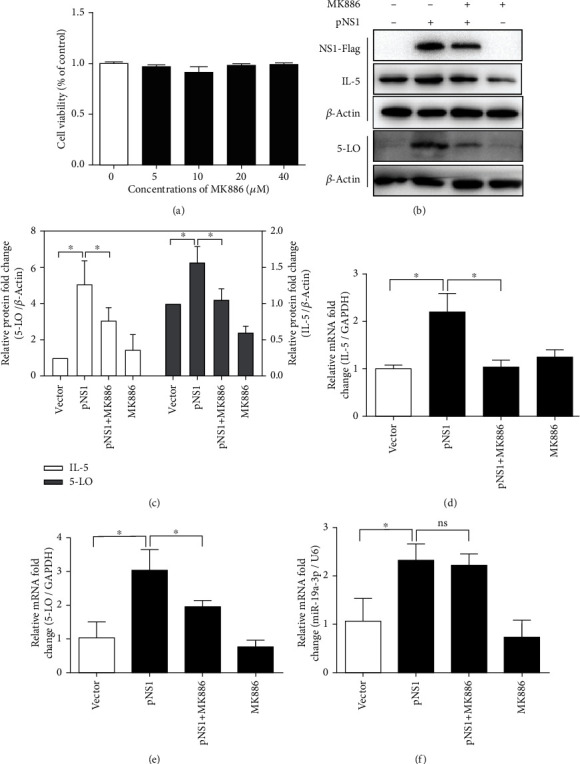
Inhibition of 5-lipoxygenase (5-LO) attenuates the promotive effects of NS1 on IL-5 but had no effect on miR-19a-3p. (a) A549 cells were treated with MK886 of different concentrations, and the cell viability was assessed after 4 hours using cell counting kit-8 assay. Data are reported as the percentage relative to 0 *μ*M. (b) Western blot analysis of 5-LO, IL-5, and NS1 protein levels in A549 cells transfected with pNS1 four hours after transfection. (c) Relative quantitative analysis of 5-LO protein and IL-5 protein, respectively. *β*-Actin served as an internal control. (d–f) The mRNA expression levels of miR-19a-3p, IL-5, and 5-LO in A549 cells transfected with pNS1 were detected by RT-qPCR four hours after transfection. U6 RNA or GADPH was used as internal control, respectively. Data are expressed as mean ± SD (*n* = 3 technical replicates). The experiment was performed three times with similar results, and one representative experiment is shown. ^∗^*P* < 0.5 vs. control group. NS1: nonstructural protein 1; pNS1: nonstructural protein 1 expressing plasmid; 5-LO: 5-lipoxygenase; IL-5: interleukin-5.

A 5-LO inhibitor MK886 was used to elucidate the effects of 5-LO on the production of IL-5, LTB_4_, and miR-19a-3p in the pNS1-transfected A549 cells. Cell viability assay and inhibition efficacy analyses showed that MK886 concentrations from 5 to 40 *μ*M had a little effect on cell viability (Figure 4(a)), while the 20 *μ*M MK886 significantly inhibited the mRNA and protein expression levels of 5-LO in the NS1-transfected cells. Therefore, the A549 cells were pretreated with MK886 (20 *μ*M) for 24 h before transfection. At 4 h after pNS1 transfection, in response to the inhibition of 5-LO, the pNS1-induced mRNA and protein expression levels of IL-5 decreased significantly (Figures 4(b)–4(e)). However, the inhibition of 5-LO did not affect the expression levels of miR-19a-3p (Figure 4(f)).

## 4. Discussion

**Figure 5:**
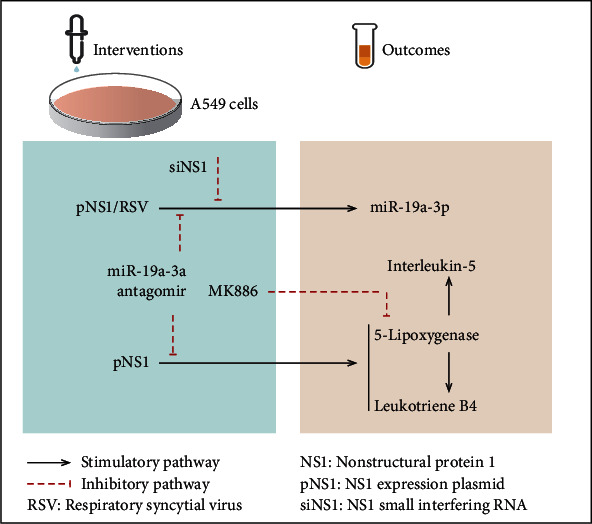
Summary of findings. RSV and pNS1 promote the expression of miR-19a-3p in A549 cells. NS1 small interfering RNA alleviates the effect. pNS1 increases the levels of 5-lipoxygenase, interleukin-5, and leukotriene B4, which could be attenuated by inhibitors of miR-19a-3p or 5-lipoxygenase. Black arrows: activation; dotted red lines: inhibition. RSV: respiratory syncytial virus; NS1: nonstructural protein 1; pNS1: NS1 expression plasmid; siNS1: NS1 small interfering RNA.

Previous reports have shown that overexpression or lack of RSV NS1 protein regulates the expression of miRNAs during RSV infection [15, 17]. The present study showed that the plasmid-mediated overexpression of RSV NS1 could regulate the 5-LO, which is a key enzyme in LT biosynthesis, and regulated the subsequent inflammatory factors such as IL-5 through miR-19a-3p, which further elucidated the complex regulatory role of NS1 protein in the pathogenesis of RSV infection (Figure 5).

The mechanism of recurrent wheezing and asthma caused by RSV infection has been widely studied [24]. RSV regulates a series of immune cells and cytokines, causing a Th2-dominated immune response and AHR [25]. In the Th2 type inflammation/allergy, the Th2 cytokines, including IL-5, which is implicated in the induction and proliferation of eosinophils, play a critical role [26]. In addition to Th2 cytokines, the LTs are also important inflammatory mediators in the pathogenesis of bronchiolitis during RSV infection [27, 28]; among which, the LTB_4_ is considered a potent chemoattractant for most of the LTs [29]. 5-LO is a key enzyme in the LT synthesis pathway, which promotes the synthesis of LTB_4_ and production of IL-5, thereby mediating the inflammatory response [30]. This study found that RSV NS1 triggered the activity of 5-LO in epithelial cells and then upregulated the expression of IL-5 and LTB_4_, which is an important pathological factor of AHR caused by RSV. Unexpectedly, the IL-5 protein levels were not consistent with mRNA levels. The level of IL-5 protein peaked at 8 hours, while the IL-5 mRNA expression increases until 24 h. A possible explanation for this discrepancy is that IL-5 protein is rapidly consumed during inflammation. Alternatively, there are other inhibitory factors that interfere with the later translation process of IL-5 mRNA. The specific mechanism still needs further study.

RSV infection can induce a wide range of miRNA expression in the host, thereby contributing to the inflammatory reactions and wheezing during the acute infectious period [18]. Among these miRNAs, miR-19a enhances the Th2 cytokine production and allergic inflammation through the regulation of cytokines and antigen receptor signaling pathways [31]. Previous studies have reported that 5-LO is a target gene of miR-19a-3p, leading to the regulation of LT functions and immune responses by regulating the 5-LO transcriptional efficiency [19]. Moreover, the production of cytokines and chemokines is also affected by the 5-LO pathway. The 5-LO inhibition can reduce the elevated inflammatory factors in lung injury [32]. In the current study, we found that the expression of miR-19a-3p was significantly upregulated in the RSV or pNS1-treated A549 cells. After inhibiting the expression of miR-19a-3p, the upregulated 5-LO expression and LTB_4_ contents were suppressed, and NS1 no longer promoted the expression of IL-5. The inhibition of 5-LO reduced the expression of IL-5 in the NS1-transfected cells but did not affect that of miR-19a-3p. These data suggest that NS1 plays an important role in the pathological formation of airway hyperresponsiveness induced by RSV and also provide a theoretical basis for targeted therapy.

There were certain limitations in this study. The effects of NS1 on the expression of miR-19a-3p were analyzed only at the cellular level and lacked further confirmation in animal models. The miRNA biogenesis can be regulated at multiple levels including transcription, processing, and modification by RNA editing [33]. There is a complicated cross-talk between miRNA synthesis and other cellular signaling pathways. RSV can also affect the biogenesis of miRNAs through the G and NS2 proteins by affecting the cellular signal transduction [34, 35]. Therefore, although the miR-19a-3p showed the same trend in NS1 inhibition experiments and NS1 plasmid experiments, the amplitude and duration of changes caused by the pNS1 and RSV were significantly different. Further studies on NS1 deletion mutation models are needed to draw further conclusions.

In summary, this study demonstrated that the RSV NS1 could regulate inflammatory factors, such as LTB_4_ and IL-5 through the miR-19a-3p and 5-LO pathway, which provided insights into the molecular mechanism of RSV in mediating asthma or recurrent wheezing.

## Data Availability

The sequence of the NS1 plasmids can be found in NCBI GenBank (accession number JQ900253.1, https://www.ncbi.nlm.nih.gov).
